# Clinician Needs and Requirements for a Decision Aid Navigator: Qualitative Study

**DOI:** 10.2196/69756

**Published:** 2025-08-21

**Authors:** Brad Morse, Carrie Reale, An T Nguyen, Erin Latella, Hannah Bauguess, Shilo Anders, Pamela Roberts, Spencer L SooHoo, Robert El-Kareh, Andrey Soares, Lisa Schilling

**Affiliations:** 1 Division of General Internal Medicine, University of Colorado Anschutz Medical Campus, 1890 North Revere Court, Aurora, CO, 80045, United States, 1 3037245138; 2 Vanderbilt University Medical Center, Nashville, TN, United States; 3 Cedars-Sinai Medical Center, Los Angeles, CA, United States; 4 University of California, San Diego, San Diego, United States

**Keywords:** qualitative research, focus groups, user-centered design, shared decision-making, decision aid

## Abstract

**Background:**

Decision aids (DAs) are important tools that support shared decision-making (SDM) between clinicians and patients, enabling patients to be more informed and engaged in decisions regarding their care. The use of DAs can increase patient knowledge, reduce decisional regret, and engage the clinician and patient in meaningful dialog. Despite proven effectiveness in enhancing patient-centered care, a gap remains in clinician use of DAs. Known clinician barriers to using DAs include (1) time constraints, (2) concerns about the match between patient need and available DAs, (3) forcing users to leave the electronic health record (EHR) to access DAs, and (4) the burden of manually entering data into the DA.

**Objective:**

This qualitative study identified the needs and requirements of clinicians to inform the design of a clinician-facing, EHR-integrated, Substitutable Medical Applications, Reusable Technologies (SMART; SMART Health IT) on Fast Healthcare Interoperability Resources (FHIR) (HL7) app, the Decision Aid Navigator (DEAN; University of Colorado Anschutz Medical Campus). The Navigator identifies and surfaces DAs that are relevant to a patient’s health care conditions (eg, atrial fibrillation), current care (eg, not on anticoagulation), and demographics (eg, check the youngest age for the stroke prevention), and facilitates documentation of SDM discussions and decisions.

**Methods:**

We conducted 13 semistructured interviews with clinicians who were recruited from 4 academic medical centers. Interviews included a demonstration of an initial, mid-fidelity, DEAN app prototype that was designed to address DA use and barriers described in the literature. The interviews focused on clinician context and use of the prototype, affordances and barriers to using the system, and clinician needs and requirements of the system. We used qualitative content analysis to code and reduce the data, using a consensus-making approach, and identify emerging themes.

**Results:**

We identified 3 overarching themes: (1) streamlined functionality may simplify workflow and decrease the burden of DA use and SDM; (2) clinicians need appropriate competencies to effectively use the Navigator and relevant DAs; and (3) trust that the Navigator suggests prevetted DAs. Unanimously, clinicians shared that the DEAN Navigator should be integrated into the EHR. To accomplish this clear priority, clinicians stated that they needed the requisite competencies to successfully use the tool within their workflow and build trust with the tool itself.

**Conclusions:**

Better tools to support and harness the benefits of SDM are needed. Overcoming the barriers of using DAs is paramount. Tools designed and developed to support DA use must be integrated into the EHR efficiently to create an opportunity for uptake of the technology by busy clinicians. If tools like DEAN can streamline the cumbersome process of documenting the use of DAs, more clinicians may potentially use DAs with their patients, given the right context and appropriate DA.

## Introduction

### Background

Shared decision-making (SDM) and the use of decision aids (DA) tools improve patient-centered outcomes, such as satisfaction with understanding the risks and benefits of health care decisions and less decisional regret [1-3]. SDM is a deliberative process and is defined as: “an approach where clinicians and patients share the best available evidence when faced with the task of making decisions, and where patients are supported to consider options, to achieve informed preferences” [4]. Some believe the SDM process is best accomplished with the use of DAs [5], which are tools specifically designed to provide easily understandable information to facilitate clinician and patient communication and understanding of information to support informed decision-making, as compared to usual care [5].

DAs often elicit a patient’s preferences and values and present evidence-based comparisons that are easily understandable to support clinicians and patients in their discussions and decision-making. A 2024 Cochrane review of 209 studies provided strong evidence that patient decision aids, DAs that target health care decisions made by patients, resulted in increased knowledge, more accurate risk perception, congruency between values and care choices, decreased decisional conflict and regret for patients, patient satisfaction with their decision, and improved communication between clinicians and patients [5,6]. In some scenarios, patients were less likely to choose major invasive surgery over conservative care and were more likely to start needed medications [6]. DAs come in many forms, including static text, videos [7], interactive websites [8], and even telenovelas [9], to support various learning preferences, cultures, and attention spans.

It is essential that patients and their care teams can work together to achieve patients’ health goals, patient-centered outcomes, and effective, high-value health care systems. As a primary process of involving patients directly in their care, SDM is explicitly supported both by the National Academy of Health (NAM) (formerly the Institute of Medicine) [10,11] and the Affordable Care Act [12,13]. For instance, Medicare includes requirements for SDM as a condition of reimbursement for implantable cardiac defibrillator [14,15], lung cancer screening [16], and the left atrial appendage closure (WATCHMAN) device placement [15].

### Barriers Facing Clinician DA Uptake

Similar to other clinical decision support (CDS) tools, DAs face many implementation barriers, such as increased workload and knowledge demands [17], and organizational factors (eg, culture and priorities) [18]. The result is the slow adoption of DAs and SDM approaches. Stacey et al found that the main barriers for uptake of DAs were (1) lack of funding to support implementation of DAs in clinical practice, including training in using DAs, (2) outdated DAs, and (3) clinician reluctance to use DAs due to increased workload, quality concerns, health record integration issues, manual data entry, and documentation burden [19,20].

Clinician DA use to support decision-making by patients is directly influenced by how cumbersome the process is to access and use the DA within existing clinical workflows [21]. Furthermore, clinicians desire a flexible approach to decision-making with patients [22]. These existing gaps in the literature suggest a need to more fully understand the complex clinical phenomenon and subjective experiences of clinicians to be better able to leverage Health Information Technology (HIT) to support expanded use of DAs.

### The 5 Rights of CDS

**Figure 1.**
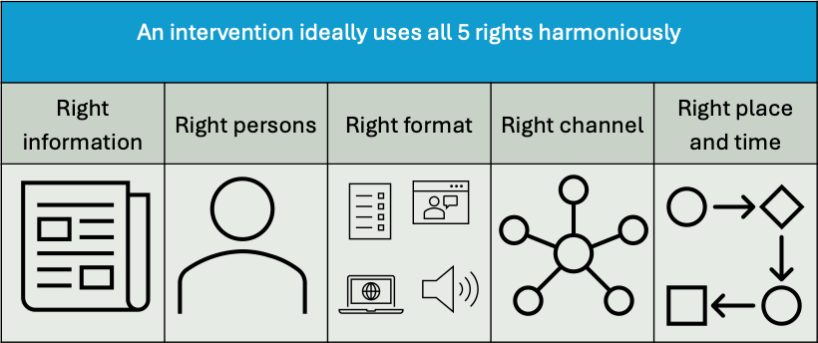
The 5 rights of clinical decision support (adapted from [23]) .

Osheroff’s “The 5 Rights of CDS,” shown in Figure 1, outline a framework for effective CDS implementation [23,24]. We applied the “5 Rights of CDS” framework to SDM support, specifically the facilitation of DA use in the SDM process. SDM and DA implementation face many of the same implementation barriers as CDS interventions. The “5 Rights of CDS” address many of the known barriers to SDM and the use of DAs. DAs provide the right information (eg, the relevant, current, and unbiased DA) to the right person (eg, clinician or patient) at the right time (eg, before the decision has been made), in the right format (eg, best format for the clinician or patient given the topic, such as written text or video and the patient’s primary language), and in the right channel (eg, within the electronic health record [EHR] patient portal).

Despite the proven benefits of DAs and SDM [6], informatics systems to address the barriers that health systems, clinicians, and patients face when attempting to use DA are lacking. The 5 Rights of CDS and the use of DA are closely aligned with the aim to improve patient care by ensuring that the right information reaches the patient or clinician at the right time. Aligning the principles of the 5 Rights and SDM can improve the quality of care and support patient engagement to make important health decisions.

Based on the documented advantages of using DAs, the 5 Rights of CDS, and the research still needed in making DAs accessible in existing workflows, the objective of this study was to understand clinician needs and requirements for a HIT solution that surfaces relevant DAs. In our proposed solution, DAs are launched in the SMART (SMART Health IT) on FHIR (HL7) [25] Shared Decision Aid Navigator System (DEAN; University of Colorado Anschutz Medical Campus) navigator app and are surfaced in the customized DEAN EHR tab. DEAN was designed and developed as an interoperable SMART on FHIR [25] app and therefore is highly applicable and generalizable.

## Methods

### Overview

We (BM) conducted semistructured interviews (SSI), while other study staff were present to take notes (EL) and assist with facilitation (AS and LS), with clinicians identified at 4 US academic medical centers across the United States using purposive sampling [26,27]. Partners at the academic medical centers identified potential interview participants. We approached individuals via email to gauge interest in participating. Eligibility requirements for the SSI included clinicians with previous DA use experience and at least 20% clinical effort. Interviews continued until we reached thematic saturation, defined as not hearing any substantive new information from research participants.

### Interview Guide and Data Collection Interaction

After a review of the literature to understand the already reported barriers and potential facilitators to DA use by clinicians, and alignment with the “5 Rights,” we (LS, AS, and BM) developed an SSI guide to elicit insight from potential future users of the Navigator and ensure the same core questions were asked of all the participants while allowing flexibility to ask probing questions on other topics that arose during the discussion (Multimedia Appendix 1).

The SSI began with an elicitation of the participants’ perspectives and experiences using DAs and having SDM discussions. This served to kick start the interview and provided the interviewer with the necessary personal context to conduct the remainder of the interview. As the purpose of the SSI was to obtain clinician input on our initial solution to the known barriers to DA use and SDM, we demonstrated a clickable, interactive prototype of DEAN (referred to as the Navigator henceforth) to demonstrate our proposed solution and collect insights from clinicians on how to further refine the prototype to support clinician use of DAs. The demonstration reviewed the known barriers and proposed solutions and facilitated input on the prototype features and functions from a user’s perspective. Each interview lasted 1 hour and was conducted on the Zoom (Zoom Video Communications Inc) [28] video conferencing platform. Sessions were recorded and professionally transcribed verbatim for analysis.

### Content Analysis Overview

**Figure 2.**
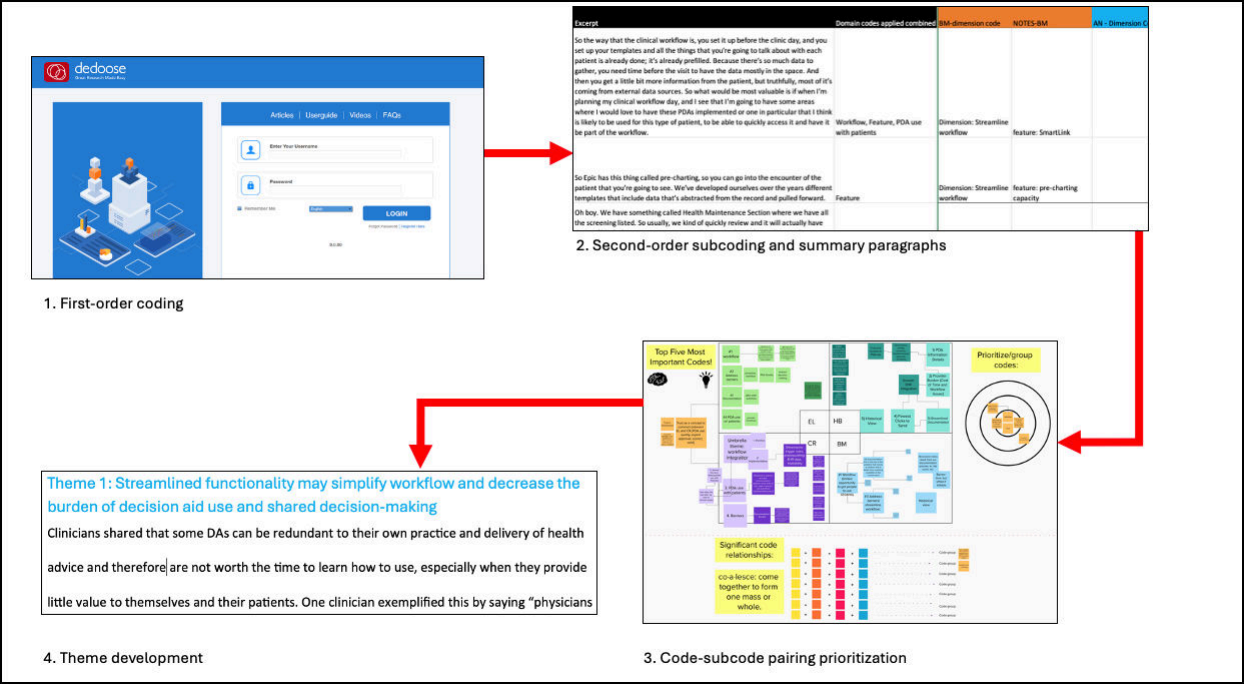
Content analysis overview.

We then conducted a 4-step qualitative coding process to analyze the interview transcripts using the Rapid and Rigorous Qualitative Data Analysis (RaDaR) technique [29-31]. This process included (1) creation and application of first-order codes, (2) application of second-order subcodes for a more nuanced and detailed coding scheme and a reduction of the code-subcode pairings to individual summary paragraphs [29,32], (3) identification and prioritization of relationships in the data, and (4) articulation of salient relationships into qualitative themes describing clinician needs and requirements of the Navigator (Figure 2).

#### Qualitative Analysis Step 1: First-Order Coding

To accomplish data reduction and thematic development, a codebook was developed that contained the initial a priori codes, ie, first-order codes [33]. We then applied the codes to the interview transcripts to identify expected phenomenon (eg, barriers to the use of DAs) [32] using Dedoose (SocioCultural Research Consultants LLC), a qualitative analysis software [34]. If emergent codes (ie, new codes) were necessary during the coding process, the team defined the emergent code and discussed adding them to the codebook on a consensus basis (agreement from all 5 team members needed for the emergent code to be added). Coding was completed by the core qualitative team (AN, CR, SA, and BM). Each transcript was coded to consensus by 2 team members [33,35,36], a process in which discrepancies are discussed until consensus is reached [37], increasing the rigor of the coding [33,35,37].

#### Qualitative Analysis Step 2: Second-Order Subcoding and Summary Paragraphs

Once all the transcripts received codes, the Dedoose excerpts were entered into an Excel spreadsheet. Coders then applied more nuanced second-order subcodes. Subcodes were applied independently to Excel excerpts by at least 2 qualitative team members, and consensus was reached (AN, CR, EL, HB, SA, and BM) [36]. Excerpts that received the same code and subcode combination were grouped together and then reduced through synthesis to a single summary paragraph for each pairing.

#### Qualitative Analysis Steps 3: Code-Subcode Pairing Prioritization

Team members (CR, EL, HB, and BM) then reviewed all the code-subcode pairing summary paragraphs to identify the most relevant pairings to the needs and requirements of clinicians to support DA use. The saliency of the code-subcode pairing was determined by the pairing’s information power [38], that is, the richness and relevance of the data in light of the study’s objective and questions. Relationships were identified between code-subcode pairings that could be used to articulate the needs and requirements of clinicians for Navigator use.

#### Qualitative Analysis Steps 4: Theme Development

Mural [39] was used to visualize relevant code-subcode pairings and to interactively coproduce themes (BM, CR, EL, and HB) that characterized and described clinician needs and requirements. We reviewed and finalized the drafted themes ensuring that they accurately reflected our data. Themes were presented to the DEAN team (LS, AS, EL, PR, SS, RE, SA, and CR) for verification of relevance to our research question.

### Ethical Considerations

The study was approved by the Colorado Multiple Institution Review Board (COMIRB #22‐1061) and adhered to all relevant ethical guidelines, including informed consent procedures. We attest to maintaining privacy and confidentiality of research subjects' data and/or identity. The project received a waiver of informed consent. We distributed a postcard consent form outlining the study to all participants prior to data collection. Participants were informed that they could stop their participation at any time. Data were anonymized. Clinicians were compensated $100 for participating in the SSIs.

## Results

### Overview

Twenty clinicians were contacted to participate in the SSIs, of which 13 participated in them, representing 4 academic medical centers, while 7 did not participate (Table 1). One of the 7 clinicians did not respond, 3 were too busy, 3 did not meet the criteria (1 had no experience with DAs and 2 did not meet the minimum clinical full-time equivalent, FTE requirement of 20%). Interviews were approximately 60 minutes long. The data collected provided data saturation for understanding the needs and requirements of clinicians interacting with the Navigator.

We identified 3 primary themes. Theme 1 describes opportunities identified by the clinicians that may support the uptake of the Navigator. Themes 2 and 3 describe needs and requirements, training and trust, which clinicians reported as necessary to use a technology like the Navigator that surfaces relevant DAs.

**Table 1.** Semistructured interview participant demographics (n=13).

Demographic variables	Participants (N=13), n (%)
Sex identity	
Female	3 (23)
Male	8 (62)
Not reported	2 (15)
Age (years)	
26‐32	1 (8)
33‐40	5 (38)
41‐49	4 (31)
50+	3 (23)
Race	
Asian	2 (15)
White	10 (77)
Not reported	1 (8)
Specialty	
Cardiology	1 (8)
Emergency Medicine	2 (15)
Family Practice	1 (8)
Geriatrics	1 (8)
Internal Medicine	5 (38)
Pediatrics	2 (15)
OB/GYN-Oncology	1 (8)
Clinical FTE^a^ (remainder of time spent on informatics)	
20%‐50%	10 (77)
51%‐100%	3 (23)

a FTE: full-time equivalent.

### Theme 1: Streamlined Functionality May Simplify Workflow and Decrease the Burden of DA Use and SDM

Clinicians shared that some DAs can be redundant to their own practice and delivery of health advice and therefore are not worth the time to learn how to use, especially when they provide little value to themselves and their patients. One clinician exemplified this by saying, “Physicians are already overburdened. Having to bring up another tool, the Navigator, and explain another decision [DA] to the patient takes more time.” Clinicians also shared that while they believed DAs can be valuable, they are not as significant an issue to tackle with their patients as there are more urgent problems the patient is experiencing.

According to the clinicians we interviewed, to combat the burdensome barriers and enable more clinicians to use DAs, the Navigator should address the following requirements: (1) integration into the EHR, (2) data collection burden reduction, and (3) documentation burden reduction. The DA delivery system that centralizes patient EHR data, populates required DA data, allows DAs to open within the EHR, and facilitates documentation of the SDM discussion in the EHR, are components that clinicians cited as desirable.

Too many clicks will be detrimental to the adoption of the DEAN technology. This concern was expressed by a clinician who asked, “If these tools exist, are they going to be within the existing workflow division or are they going to add extra clicks?” Another clinician warned, “All the third-party vendors try to make it seem like it’s seamless. It never is. And busy clinicians don’t fall for the seamless talk,” expressing the importance of immediately and consistently demonstrating EHR integration in the existing workflow. Several clinicians recognized that automating tasks (eg, documentation) may increase time in the visit for patient engagement and enhanced patient-clinician relationships, sharing that “The clinician should spend time catching up with the patient, asking how they’re doing and having that human aspect to it.”

To overcome common barriers, it is not only important that the Navigator is integrated into the EHR, but that the Navigator documentation process be integrated within the EHR note-writing function to make it as easy as possible. Automated note population of DA use with patients was strongly preferred. Some physicians stated they document their use of the DA, including discussing risks with the patients and what they and the patient decided together. “The report, that would then populate into the note, which I think would be brilliant and is a perfect way to include it in the template.” However, some find it difficult to balance thorough notes that are simple for patients to understand. In addition, some physicians find it challenging to either write a note with the patient during the visit (which can be very time-intensive) or to remember enough about the visit to write about it later. Templates, or even having the patient come in already engaged with the DA by entering required data, make the documentation process easier for clinicians.

### Theme 2: Clinicians Need Appropriate Competencies to Effectively Use the Navigator and Relevant DAs

The use of a DA delivery system, like the Navigator, to support SDM involves interaction with complex technology and health care information. Clinicians communicated that training would be important before using the Navigator. They also felt they needed the knowledge and skills necessary to use the DAs. For clinicians, this includes the training required to access appropriate DAs in the Navigator and use the Navigator within the fast-paced and time-pressured clinical workflows, as well as possessing sufficient knowledge and experience with the clinical content of a DA. Currently, clinicians report being overburdened and learning how to use the Navigator and various DAs to implement the DSM processes into their workflow would be time-consuming. Many clinicians shared that they see many patients in a day and learning a new tool like the Navigator would only add to their busy schedules.

Clinicians identified the need to determine whether a DA is well designed and evidence-based. Alternatively, clinicians would rely on others (eg, a health system governing group or a medical organization) to endorse the DA. Clinicians described the substantial effort expended to adopt a new DA and stay current on the underlying evidence base. Evidence can change rapidly or be based on studies that are unfamiliar to the clinician. “I have very deep knowledge of the CDS tools that I use and understand how well they were validated and who did the studies and how high quality the studies were…we do journal clubs to talk about these tools…There is a depth of understanding and trust that happens before we apply them in clinical practice.” Another clinician added, “Some of the other barriers that come up are comfort with the data. Like, you must know the material well yourself to be able to use some of these…and it changes all the time, so you must really be up to date.”

Clinicians indicated that competency with the Navigator and specific DAs could be gained through trial-and-error adaptive learning. The opportunity to learn through practice was seen as a strategy to gain the experience and knowledge required to comfortably integrate DAs into clinical workflows. “I think trialability, (eg, using the tool before having to use it with patients) is another way to get over the mental model of adoption.” Trialability will add work to the busy clinician’s schedule, however, “And if you just gave me a new tool (DA and the Navigator) and say, hey, this is good for head injury patients, I would say, all right, I’ll read about it after shift, and I may apply to the future.” Relatedly, some clinicians highlighted the Navigator’s ability to increase their competence and knowledge, stating, “there’s a lot of times when not only am I having trouble explaining a complicated decision to a patient, but there’s also actually information in these tools [the relevant DA] that I don’t know.”

### Theme 3: Clinicians Must Trust the Navigator to Implement Use in Their Standard Workflow

A common response to the tool demonstration was that trust in the Navigator is a crucial prerequisite for the successful adoption of DAs. Clinicians are often reluctant to adopt technologies, such as the Navigator, when their benefits are uncertain [40]. Two types of trust were identified by clinicians.

#### Trust That the Navigator Is Aligned With Clinic Workflow

One clinician recognized the importance that streamlining the busy and burdensome clinical workflow may have on clinician’s assessment of the Navigator. Trusting that the Navigator works smoothly within the busy clinical workflow is important because an indirect patient assessment of both the clinician and Navigator may result in, “…we’re dealing with time and trust” of the patient. Violation of this expectation could erode clinician trust in the Navigator. Another clinician recognized that the Navigator might streamline the use of DAs, but gaining personal experience with the tool is an essential component for building trust in the system, “…I think after you try these three or four times, then the mental model barrier goes away. You’re like, ‘Oh, this is kind of helpful. It’s not usurping me.’”

#### Trust That the Navigator Suggests Prevetted DAs They Can Use

Finally, selecting a quality DA and becoming familiar enough with its content to use it effectively during a visit was a concern for clinicians. Physicians feel that they need to establish familiarity with and trust in a specific DA in advance of using the DA during a patient encounter. One clinician shared, “If there were a lot of these [DAs] and I wasn’t personally familiar with them, deeply familiar, then I would have difficulty…trying to make sure that I’m comfortable that this information applies to their particular case.” Clinicians felt that while information embedded in the Navigator about a DA, such as evidence base or journal citations, could support clinicians’ comfort with using a DA, clinicians would still have to become familiar with the DA to trust it enough to put it into practice. However, clinicians stated that if DAs were prevetted by subject matter experts or committees at the local institution, uptake would be easier because the institution had previously endorsed veracity and use of the DA.

## Discussion

### Principal Findings

Our findings in Theme 1 reveal that clinicians have concerns about their ability to use DA and have SDM discussions during routine clinical care due to visit length time constraints, which are further impacted by technology that does not fit within their workflow and the need to address the most pressing and medically serious issues during visits. Workflow-concordant technology is essential for adoption and uptake, and clinicians stressed the importance of seamless EHR integration, prevetted and suggested DAs, DAs that do not require duplicate data entry, and the facilitation of the documentation of SDM discussions. While every concern matters, and clinicians and patients should work together to prioritize the visit agenda, clinicians are trained to recognize signs of urgent or life-threatening conditions that must take precedence. For instance, addressing symptoms of a possible heart attack would understandably take precedence over discussing sleep difficulties, regardless of the existence of the perfect insomnia management DA. Clinicians also expressed concerns about redundancy and a lack of value to DAs due to a clinician’s expert knowledge of a topic or a simplistic decision. Many clinician recommendations and patient decisions do not require use of a DA, such as the recommendation and decision to take antibiotics for confirmed bacterial pneumonia. This contrasts with the high-stakes decision to have an implantable cardiac defibrillator, which is highly dependent on a patient’s desire to lengthen their life and the distress caused by expected and realized electronic shocks. It is an obvious and understandable stance that DAs should only be used when they provide added value to complex SDM discussions. Although research suggests that clinicians recognize that they use paternalistic methods more often than they would prefer and that they have more limited discussions than they feel are optimal, mainly focusing on treatment options but not comparative pros and cons, and patient values and preferences. Many patients would like to be more engaged in their health care decisions [41,42].

Our findings formulated in Theme 2 indicate that clinicians may need to acquire new skills and knowledge to use a tool such as the Navigator and to effectively integrate DA use into existing health care workflows. In a study by Abbass et al [43], who found that lack of appropriate training was a key barrier to technology use, our research participants also commented on the need for both Navigator training and time to become familiar with a DA’s content before using it with patients. A qualitative analysis of expert interviews in the context of artificial intelligence [44] described core competencies that clinicians need to effectively use these types of advanced technological tools within their clinical workflow [44]. The competencies identified include basic knowledge about the purpose and application of the tools; how to evaluate the quality, accuracy, and contextual appropriateness of the tools; how to adapt to changes in roles and workflows resulting from implementation; and the need to participate in continuing education related to use.

Similar competencies will be required for DA use in SDM, and with a tool like the Navigator, implementation planning and system training curriculum should account for the additional learning needed to successfully operationalize DAs in real-world settings. When clinicians participate in education opportunities on DAs, they are more likely to share a DA with a patient and engage in formal SDM [45]. With an increased skill set, it may follow that patient satisfaction with DAs and the SDM process would also improve. However, due to the high potential for ongoing changes to DA content, as new evidence and care standards emerge, continuing education will also be required. Continuing education should focus on SDM and DAs as a teachable skill [46].

As explained in Theme 3, while the benefits of DAs have been documented, the lag in widespread “trust” will persist until we can address multiple barriers. When designing and developing HIT that promotes trust, Jones et al [47] recommend designers clearly explain how the technology works, involve end-users in the development of the HIT, provide training and support for targeted end-users, allow for customization by end-users so that they can tailor the way they use the technology, and develop feedback mechanisms so that designers and developers can address system issues [47]. In our interviews, clinicians highlighted issues consistent with the recommendations made by Jones et al [47]. They wanted to trust that the Navigator system and the process of its implementation would work in their existing workflow. Therefore, before clinicians can trust the Navigator, which is mandatory, they need to be assured that the Navigator meets their needs and requirements as outlined in Themes 1 and 2.

The goal of health care technology is to provide greater convenience and clinical task efficiency, yet Abbas et al[43] cite key findings that barriers exist at technological, individual, and institutional levels that prevent widespread trust and adoption of health care technology. Specifically, these findings highlight compatibility, reliability, usability, training, and governance as barriers designers need to overcome to build clinician trust in new technologies [43]. Concerns were shared in the interviews that the Navigator would be vulnerable to these same trust issues and would need to be addressed before uptake.

The results of this study informed the future design, evaluation, and implementation plans of our proposed DEAN system. The rich qualitative data confirmed many of the functional requirements our team had previously identified based on known barriers to DA use and SDM. It also allowed further specification of some functional requirements and advanced the prototype to be used in further user-centered design sessions. For example, we sought to ensure the Navigator interface would integrate with the EHR in a manner consistent with EHR use in clinical care, opening within the EHR and not within an external browser. We also confirmed the need to create a system to support institutional curation of vetted DAs. In response to clinician input, we designed a system that surfaced Suggested DAs based on patients’ current problems and medications, in a manner that was noninterruptive, as clinicians expressed that DA use would be based on agenda prioritization and the need for a formal SDM discussion and the added value of the DA to the discussion. We also planned to streamline the filing of the SDM discussion note to the EHR through a variety of commonly used pathways. Our hope is that data-informed improvements in DA use workflows through the DEAN system, including the Navigator, will support greater uptake of DAs and enhance SDM conversations for clinicians and patients.

Furthermore, we believe the Navigator could lead to more trust on the part of the clinician. For example, the Navigator is designed to have a tile in the interface for each DA. Each DA tile will have an information button that provides readily available information on topics such as the references for the evidence on which the DA was developed and the DA creators. The information button also provides information to indicate the governing person or committee at the institution responsible for vetting and releasing the DA within the Navigator. Finally, we believe that training in Navigator use and time for clinicians to become familiar with the DAs will increase clinician trust of the tool.

### Limitations

Some participants conflated the idea of risk calculators with DAs, but the facilitators were able to clarify the difference and provide relevant examples. We only conducted interviews with academic clinicians and community clinicians; practices may have different opinions. If anything, we suspect their time constraints and need to focus on revenue-generating activities may be even greater for community physicians. This could lead to varying stances, such as a clinician never making the time to use a DA and participate in a thorough SDM discussion, or a clinician who welcomes the timesaving and workflow-aligned functionality that the Navigator offers.

### Conclusions

Future directions include the further development of the DEAN system, including the Navigator, and other backend systems to support DA curation and documentation of key facts such as DA governing entities, DA authors, evidence citations, evidence in the form of brief written summaries, and DA version date, including when the DA was last updated. Our hope is that the process of including clinicians early in the process of the DEAN system design leads to a tool that is workflow aligned and addresses the major barriers associated with DA use and SDM discussions. This should result in a system that has greater uptake and adoption, ultimately leading to better patient engagement and satisfaction with their medical decisions.

## Supplementary material

10.2196/69756 Multimedia Appendix 1 DEAN: Semistructured interview guide for Navigator requirements.

## Abbreviations

CDS: clinical decision support
DA: decision aid
DEAN: Decision Aid Navigator
EHR: electronic health record
FHIR: Fast Healthcare Interoperability Resources
HIT: Health Information Technology
NAM: National Academy of Health
PDA: patient decision aid
RaDaR: Rigorous and Accelerated Data Reduction
SDM: shared decision-making
SSI: semistructured interview
