# Supplementary material for: Clinician Needs and Requirements for a Decision Aid Navigator: Qualitative Study
Source: JMIR Hum Factors. 2025 Aug 21;12:e69756. doi: 10.2196/69756 (PMC12370259; doi:10.2196/69756)
Supplement: Multimedia Appendix 1 [file humanfactors-v12-e69756-s001.docx]

SEDANS: Semistructured Interview Guide

# Navigator Requirements: for HCP personnel

Purpose: To support clinicians and patients in finding and using PDAs that are relevant to the patient’s health and the decisions they make regarding their health, such as whether to have a certain procedure or whether to start or adhere to a certain medication.

Participants: Front-line clinicians (HCPs) [clinician, clinical informaticians]

Method: Semistructured interview (SSI)

Introductory script:

Greetings. My name is ____ and I am a research team member from the University of Colorado Anschutz Medical Campus.

We are funded to build a HIT system that addresses the barriers that clinicians have in using Patient Decision Aids (PDAs). Use cases include shared use of a PDA with a patient, clinician-only use, or patient use before/after the medical appointment, among others. Some examples of PDAs we will refer to during this discussion are listed below, beginning with one for helping patients and clinicians decide on a treatment for hypertension. You can see it on my shared screen now (Pick one of the PDAs below to share on-screen):

1. https://www.healthdecision.org/tool#/tool/hypertension

The hypertension PDA allows entry of data specific to the given patient, provides risk calculations, the likelihood of meeting a certain goal with various treatment options, and the benefit of meeting the goal –which is reducing future cardiovascular risk.

Other PDA examples we may want to discuss include the Statin Choice Decision Aid, the Implantable Cardioverter Defibrillator (ICD) and the one required for Medicare coverage of low-dose Lung CT for lung cancer screening in current and former smokers- documented use of a shared decision-making discussion is required for Medicare coverage, and this is best done using a PDA.

1. https://statindecisionaid.mayoclinic.org
2. https://patientdecisionaid.org/icd
3. https://www.thoracic.org/professionals/clinical-resources/disease-related-resources/decision-aid-for-lung-cancer-screening-with-ct.php

As you know, clinician barriers to PDA use include having to go outside the EHR to find and use PDAs; duplicate data entry, although not all PDAs require data entry; finding a high-quality and relevant PDA; documenting the shared decision-making discussion that occurred with the patient.

We were funded to build a system to address these barriers. We will be applying the “5 Rights of Clinician Decision Support” to Shared Decision-Making Support where a PDA should be used with the right person, provide the right information, use the right intervention format and the right channel, at the right time in the workflow. Our system has two major components: a Navigator System that is integrated with Epic and supports the “5 Rights” for patients and clinicians, and an Administrative Portal that allows HIT/CI personnel to maintain the information required to support the Navigator System. Because these two components are tightly aligned, we are going to ask for your input on both.

Guided Discussion:

- Tell me a bit about your use of PDAs?
- What are your greatest barriers to using PDAs with patients?
- What would help address these barriers?
  - Plan to discuss each barrier…
    - Finding the right PDA
    - Knowing it is a high-quality PDA
    - Using the PDA with patient having as shared decision-making discussion
    - Duplicate data entry
    - Documenting shared decision conversations and decisions.
- What are the best ways to use PDAs with patients? Do you think in some circumstances a PDA could be used by a patient between visits (pre/post), in preparation for a discussion/decision, and in some circumstances might be best used together with a clinician?
- What role, if any, does the patient play in the documentation process? What would you want the patient to document?
  - Please share an example of a PDA you've used in the past, including what information did you include in your clinical note about using it.
  - What difficulties do you currently experience with documenting your use of PDAs, if any?
- What information do you want about the PDAs aside from the name of the PDA that would help you know whether to use it or not with a given patient?
  - Why?
  - How?
  - What information is important for you to know about a PDA before you decide to use it in your clinical practice?
  - If you were to recommend a specific PDA to colleague, what information would you tell them about it?
- Tell us if a historical view of a patient’s past viewed/used PDAs would be helpful and why.
  - How do you currently keep track of the PDAs you've used in the past with a particular patient? What is difficult about the current way you keep track of PDAs you've used in the past?
  - What would make it easier for you to keep track of PDAs you've used with a patient in the past?
- We would like to follow up on something earlier that you shared.
- Is there anything else you would like to tell us before we conclude?
